# Potential contribution of the uterine microbiome in the development of endometrial cancer

**DOI:** 10.1186/s13073-016-0368-y

**Published:** 2016-11-25

**Authors:** Marina R. S. Walther-António, Jun Chen, Francesco Multinu, Alexis Hokenstad, Tammy J. Distad, E. Heidi Cheek, Gary L. Keeney, Douglas J. Creedon, Heidi Nelson, Andrea Mariani, Nicholas Chia

**Affiliations:** 1 Department of Surgery, Mayo Clinic, Rochester, MN 55905 USA; 2 Department of Health Sciences Research, Mayo Clinic, Rochester, MN 55905 USA; 3 Department of Obstetrics and Gynecology, Mayo Clinic, Rochester, MN 55905 USA; 4 Department of Laboratory Medicine and Pathology, Mayo Clinic, Rochester, MN 55905 USA; 5 Center for Individualized Medicine, Mayo Clinic, Rochester, MN 55905 USA; 6 Present Address: North Memorial Medical Center, Robbinsdale, MN 55442 USA

**Keywords:** Microbiome, Endometrial cancer, Uterus, 16S rDNA, *Porphyromonas*, *Atopobium*

## Abstract

**Background:**

Endometrial cancer studies have led to a number of well-defined but mechanistically unconnected genetic and environmental risk factors. One of the emerging modulators between environmental triggers and genetic expression is the microbiome. We set out to inquire about the composition of the uterine microbiome and its putative role in endometrial cancer.

**Methods:**

We undertook a study of the microbiome in samples taken from different locations along the female reproductive tract in patients with endometrial cancer (*n* = 17), patients with endometrial hyperplasia (endometrial cancer precursor, *n* = 4), and patients afflicted with benign uterine conditions (*n* = 10). Vaginal, cervical, Fallopian, ovarian, peritoneal, and urine samples were collected aseptically both in the operating room and the pathology laboratory. DNA extraction was followed by amplification and high-throughput next generation sequencing (MiSeq) of the 16S rDNA V3-V5 region to identify the microbiota present. Microbiota data were summarized using both α-diversity to reflect species richness and evenness within bacterial populations and β-diversity to reflect the shared diversity between bacterial populations. Statistical significance was determined through the use of multiple testing, including the generalized mixed-effects model.

**Results:**

The microbiome sequencing (16S rDNA V3-V5 region) revealed that the microbiomes of all organs (vagina, cervix, Fallopian tubes, and ovaries) are significantly correlated (*p* < 0.001) and that there is a structural microbiome shift in the cancer and hyperplasia cases, distinguishable from the benign cases (*p* = 0.01). Several taxa were found to be significantly enriched in samples belonging to the endometrial cancer cohort: Firmicutes (*Anaerostipes*, *ph2*, *Dialister*, *Peptoniphilus*, *1–68*, *Ruminococcus*, and *Anaerotruncus*), Spirochaetes (*Treponema*), Actinobacteria (*Atopobium*), Bacteroidetes (*Bacteroides* and *Porphyromonas*), and Proteobacteria (*Arthrospira*). Of particular relevance, the simultaneous presence of *Atopobium vaginae* and an uncultured representative of the *Porphyromonas* sp. (99 % match to *P. somerae*) were found to be associated with disease status, especially if combined with a high vaginal pH (>4.5).

**Conclusions:**

Our results suggest that the detection of *A. vaginae* and the identified *Porphyromonas* sp. in the gynecologic tract combined with a high vaginal pH is statistically associated with the presence of endometrial cancer. Given the documented association of the identified microorganisms with other pathologies, these findings raise the possibility of a microbiome role in the manifestation, etiology, or progression of endometrial cancer that should be further investigated.

**Electronic supplementary material:**

The online version of this article (doi:10.1186/s13073-016-0368-y) contains supplementary material, which is available to authorized users.

## Background

The causative or triggering agents for endometrial cancer remain elusive despite continued research along the PI3K/PTEN/mTOR/HIF axis in type I [1] and the p53 tumor-suppressor system in type II endometrial cancer [2]. Host genetics explain only 20 % of endometrial cancer incidence through microsatellite instability (MSI) [3] or abnormalities in aerobic glycolysis [4]. The efforts to identify the cause of the remaining 80 % of cases have led to studies of a number of environmental and host factors including hormones [5], obesity [6], and diabetes [7]. However, these alone do not address the question of tumorigenic mechanism. There is a need to examine potential causative agents, studies of which bring the promise of developing targeted prevention strategies.

Here, we explore a major source of environmental influence on the uterine microenvironment—the microbiome. Microbial influence on the etiology and progression of cancer has already been well established for *Helicobacter pylori* and gastric cancer [8]. Recent high-throughput sequencing assays have revealed associations between colorectal cancer and infection with *Fusobacteria* [9] and *Porphyromonas* [10] that are suggestive of a broader microbiome role in cancerous processes. Like the two examples above, endometrial cancer also often arises from a pro-inflammatory profile [11]. We sought to explore the potential microbial triggers for inflammation and tumorigenesis through examination of the uterine microbiome in participants with endometrial cancer.

The microbial partners along the female reproductive tract have been long known to play an important role in health and disease along the woman’s reproductive tract. Lactic acid producing microbes have a strong role in determining the microbial community membership of the vaginal microbiome and have been shown to protect against infection [12]. Gynecologic pathogens associated with bacterial vaginosis, such as *Atopobium vaginae* and *Gardnerella vaginalis* have been associated with obstetric complications, such as preterm labor [13]. However, few studies have directly probed the microbes within the uterine environment and how these microbes could influence cancer within the endometrial lining. Given the inflammatory profile in endometrial cancer manifestation, we hypothesized that there is a microbiome component in the malignancy and that its signature in patients diagnosed with the disease is distinguishable from that of patients without malignancy.

## Methods

### Participant enrollment

We report the results from 31 participants enrolled at the Gynecologic Division, Mayo Clinic, Rochester, MN under an IRB approval protocol (12–004445). The inclusion criteria were the following: 18 years of age or older; women undergoing hysterectomy by any standard surgical approach; undergoing hysterectomy for benign disease, hyperplasia, or any stage of endometrial cancer. Patients with any of the following criteria were excluded from our study: women who were pregnant or nursing; had taken antibiotics within two weeks preceding surgery; surgeon using morcellation during the hysterectomy procedure, due to the size of the uterus or for any other reason. Upon enrollment the participants were requested to fill out an optional questionnaire about sexual and reproductive health and history. The metadata from the questionnaires was stored at REDCap [14]. Cancer participants were also requested to provide a stool sample for the search for putative endometrial cancer signatures.

### Sample collection

#### Vaginal and cervical samples

All participants were requested not to douche with betadine on the day of surgery or the day immediately preceding it. All the vaginal and cervical swabs and scrapes were collected by the surgeon (with guidance on site by the research team) immediately after the administration of anesthesia and immediately preceding the standard pre-surgical betadine douche. Both the vaginal and cervical swabs were performed with three sterile Dacron swabs each and placed in a sterile tube with 1 mL of Tris-EDTA (TE) buffer kept on dry ice until storage at –80 °C. One of the vaginal swabs was used for immediate on-site vaginal pH measurement with a Hydrion measuring pH tape. The scrapes were performed using sterilized (autoclaved at 121 °C for 20 min) pap smear spatulas and placed in sterile tubes with TE buffer kept in dry ice until storage at –80 °C.

#### Uterine, Fallopian, and ovarian samples

Once removed, the uterus, Fallopian tubes, and ovaries were handed by the surgeon to the instrumentalist nurse who placed them inside a sterile transport bag and into a closed sterile container. The research team then transported the container to the pathology lab (within the same clean area) where the organs were handed to a pathologists’ assistant (PA) to be processed under sterile conditions. The grossing station where the specimen was processed was sterilized by the research team, including all the tools needed by the PA for handling. The PA used surgical gloves and mask when handling the specimen. The PA performed a bilateral cut of the uterus and splayed it. The research team advanced to the collection of the uterine swabs (Dacron) and scrapes (sterilized pap smear spatulas) and documentation (by placement of push pins in sampled locations and digital photograph). The PA then proceeded to the aseptic collection of samples needed for the diagnosis and, once complete, the research team collected the uterine, Fallopian, and ovarian biopsies (approximately 4 mm of tissue was collected per biopsy by the use of a pair of sterile tweezers, scalpel, and surgical ruler). Each collected sample was placed in a sterile tube with 1 mL of TE buffer and kept on dry ice until storage at –80 °C. A petri dish with Lysogeny broth (LB) was kept open on the grossing station during sample collection to detect any possible airborne contamination of the specimen. The LB was swabbed and the swab was stored in a tube with 1 mL of TE and kept on dry ice until storage along with all the other samples.

### Sample processing

Once thawed, the swab and scrape samples were vortexed to bring the collected material into solution. The biopsy samples were macerated by the use of sterile pestles. The swab and scrape samples were centrifuged for 10 min at 10,000 *g* to collect the bacterial cells and the supernatant was discarded. All genomic DNA extractions were performed by using the MoBio PowerSoil Kit (MoBio Laboratories, Inc., Carlsbad, CA, USA) as described by the manufacturer; however, instead of vortexing, an MP FastPrep (MP Biomedicals, Solon, OH, USA) was used instead, for 60 s at 6.0 m/s, to obtain a more effective and rapid lysis of the cells. After extraction the DNA content was measured using High Sensitivity Qubit (Life Technologies Corporation, Carlsbad, CA, USA). The V3-V5 region of the 16S rDNA was then amplified through a polymerase chain reaction (PCR) as follows: 25 μL of Kapa HiFi (Kapa Biosystems, Woburn, MA, USA), 1.5 μL (10 uM) forward primer, 1.5 μL (10 uM) reverse primer, 50 ng of DNA with the remaining volume being added by molecular grade water (up to a final volume of 50 μL per reaction). The forward primer was the universal primer 357 F (5’GTCCTACGGGAGGCAGCAG3’) with the added construct on the 5’ end of the 5’ Illumina Adapter (5’AATGATACGGCGACCACCGAGATCTACAC3’) + Forward Primer Pad (5’TATGGTAATT3’) to a total sequence: 5’AATGATACGGCGACCACCGAGATCTACACTATGGTAATTGTCCTACGGGAGGCAGCAG3’ and the universal bacterial reverse primer was 926R (5’CCGTCAATTCMTTTRAGT3’) with an added construct on the 5’ end of the reverse complement of 3’ Illumina adapter (5’CAAGCAGAAGACGGCATACGAGATGCCGCATTCGAT3’) + Barcode (12 base pairs) to a total sequence: 5’CAAGCAGAAGACGGCATACGAGATGCCGCATTCGATXXXXXXXXXXXXCCGTCAATTCMTTTRAGT3’. The barcode introduced in the reverse primer construct was unique to each sample, functioning as a genetic ID for sequencing. The PCR cycle was the following: 95 °C for 3 min, 98 °C for 20 s, 70 °C for 15 s, 72 °C for 15 s, cycle repeated 34 times, and 72 °C for 5 min. The products of the amplification were verified by a TapeStation D1K Tape (2200 TapeStation Instrument, Agilent Technologies, Santa Clara, CA, USA) to be free of contamination and to contain the expected amplification size, approximately 700 base pairs. If the amplification was unsuccessful, the parameters of the reaction or cycle were adjusted in repeated attempts. In some cases (mostly biopsy samples) the amplification was not successful even after repeated attempts. The reduced number of microorganisms present in the upper reproductive tract is likely to justify this result and attests for the success of the sterile collection of the samples. In samples that failed 16S rDNA amplification, NEBNext Microbiome DNA Enrichment Kit (New England Biolabs Inc., Ipswitch, MA, USA) was used to separate the microbiome from the human DNA to increase the odds of a successful amplification from samples naturally enriched with human DNA (mostly tissue samples). Controls of both the DNA extraction and Microbiome Enrichment processes were performed and are shown in Supplement 5. Upon verification the PCR products were purified using Agencourt AMPure (Beckman Coulter, Brea, CA, USA). After purification the concentrations were measured using Qubit High Sensitivity. The 16S rDNA sequencing was performed by the MGF (Medical Genome facility at Mayo Clinic, Rochester) using a high-throughput next-generation Illumina MiSeq (San Diego, CA, USA) sequencing platform.

### Sequence analysis

Sequence reads were aligned with our own custom multiple alignment tool known as the Illinois-Mayo Taxon Operations for RNA Dataset Organization (IM-TORNADO) that merges paired end reads into a single multiple alignment and obtains taxa calls [15]. IM-TORNADO then clusters sequences into operational taxonomic units (OTUs) using AbundantOTU+ [16].

### Sequencing outcome

A total of 16,366,472 sequence reads (17,657–828,181 reads per sample) were obtained (mean of 199,591 ± 190,153 reads) after quality control. Further processing for visualization was performed using QIIME [17] and METAGENassist [18].

### Data analysis

#### α-diversity and β-diversity analysis

To compare the microbiota composition between cohorts, we summarized the data using both α-diversity and β-diversity. α-diversity reflects species richness and evenness within bacterial populations. Two α-diversity metrics, the observed OTU number and the Shannon index, were investigated. Rarefaction curves were used to compare the α-diversity measures. The observed OTU number reflects species richness, whereas the Shannon index measures both species richness and evenness. β-diversity reflects the shared diversity between bacterial communities in terms of ecological distance between samples; different distance metrics provide distinctive views of community structure. Two β-diversity measures (unweighted and weighted UniFrac distances) were calculated using the OTU table and a phylogenetic tree (“GUniFrac” function in the R package GUniFrac) [19]. The unweighted UniFrac reflects differences in community membership (i.e. the presence or absence of an OTU), whereas the weighted UniFrac captures this information and also differences in abundance. Rarefaction was performed on the OTU table before calculating the distances.

To assess the association with α-diversity, we fitted a linear mixed effects model (LME) to the α-diversity metrics with a random intercept for each subject (“lme” function in R package “nlme”), adjusting for covariates if necessary. Wald test was used to assess the significance. To assess the association with β-diversity measures, we used a variant of PERMANOVA procedure (“adonis” function in the R “vegan” package), which is a multivariate analysis of variance based on distance matrices and permutation [20]. To retain the within-subject correlation, we used a block-permutation scheme, where samples from the same participant were assigned a different subject ID. Significance was assessed by 1000 permutations and the covariate was adjusted if necessary. Ordination plots were generated using non-metric multidimensional scaling (NMDS) as implemented in R (“metaMDS” function in the R “vegan” package).

To test for the correlation between organs, we used a permutation test based on Bray-Curtis distance with the test statistic calculated as the distance between the organs from different participants minus the distance between the organs from the same participant. We next permuted each participant for the same organ type using the same block-permutation scheme as above. The *p* value was calculated as the percentage of permutations that produce a test statistic more extreme than what is observed. To identify the taxa shared by both organs, we used a taxon-specific Euclidean distance, defined based on the presence and absence of a given taxon, and applied the same permutation test. To test whether the distance from cohort 1 to cohort 2 is greater than the distance from cohort 1 to cohort 3, we used a permutation test with the test statistic as the difference between these two distances and block-permutation was used for assessing the significance.

#### Differential abundance analysis

We conducted differential abundance analysis at phylum, family, and genus levels and filtered rare taxa with prevalence less than 20 % to reduce the number of the tests. We fit a generalized mixed-effects model to the taxa count data using the PQL method, assuming a random intercept for each participant to account for within-subject correlation (“glmmPQL” in R “MASS” package). We fitted an overdispersed Poisson to the counts if the zero proportion is less than 25 % and an overdispersed Binomial model (presence/absence) otherwise. For the overdispersed Poisson model, we included the log of library size as an offset to account for variable sequencing depth. In the overdispersed Binomial model, the log of library size was included as a covariate to account for potential dependence of occurrence probability with sequencing depth. We used the winsorized data (97 % upper quantile) to reduce the potential impact of outliers upon the parameter estimates. To improve power to detect differential taxa, which show consistent change in both the uterus and lower tract microbiome, we pooled the uterus and lower tract data and included the sampling site (uterus/lower tract) as a covariate in the model. The same analyses were also repeated for both datasets separately to confirm the source of the identified signals using pooled data. Statistical significance was assessed based on the Wald test. False discovery rate (FDR) control (B-H procedure, “p.adjust” in standard R packages) was used for correcting for multiple testing, and FDR-adjusted *p* values or q values will be reported. All statistical analyses were performed in R 3.0.2 (R Development Core Team, Vienna, Austria). The receiver operating characteristic (ROC) curve and area under the curve (AUC) were generated using the median of the replicates with the software generated by Johns Hopkins. (http://www.rad.jhmi.edu/jeng/javarad/roc/).

## Results

### Participant population

A total of 31 Caucasian patients undergoing hysterectomy were included in this study. Of those, ten women were diagnosed with a benign gynecologic condition (control cohort), four women were diagnosed with endometrial hyperplasia (cancer precursor, hyperplasia cohort), and 17 women were diagnosed with endometrial cancer (cancer cohort). All diagnoses were made based on the final surgical pathology following hysterectomy. Healthy, asymptomatic women were not included in our study because hysterectomies (surgical removal of the uterus) are not performed on healthy individuals. The inclusion of this population in our study would mandate a different collection protocol to assess the uterine environment and involve the inclusion of multiple confounding variables that could influence the microbiome data. Nevertheless, the inclusion of a variety of benign uterine conditions in our control group provides an assessment of the microbiome that is specifically associated with a cancerous condition and not simply the result of a diseased state. Patients diagnosed with endometrial cancer were significantly older, predominantly postmenopausal, and hypertensive (Table 1).

**Table 1:** Patient demographics

Variables	Benign (n = 10)	Cancer (n = 17)	*p* value	Hyperplasia (n = 4)	*p* value vs. benign	*p* value vs. cancer
Age (years) – Median, IQR	44.5 (42.5–52.5)	64 (58–71)	0.0001	54 (50.75–62.5)	0.0552	0.08
Caucasian ethnicity (%)	10 (100)	17 (100)		4 (100)		
BMI – Median, IQR	26.6 (23.8–34.1)	32.1 (26.8–40.2)	0.07	35.4 (24–40.8)	0.29	0.89
Menopausal status			0.0034		>0.99	0.0526
Pre/Peri	8	3		3		
Post	2	14		1		
Gravida – Median, IQR	2 (2–3.25)	1.5 (0–4)	0.666	0 (0–2.25)	0.1	0.19
Parity – Median, IQR	2 (2–3)	1.5 (0–4)	0.569	0 (0–2.25)	0.13	0.23
History of hypertension			0.0362		0.85	0.31
Yes	1	10		1		
No	8	7		3		
Unknown	1	0		0		
History of diabetes			0.621		>0.99	0.54
Yes	1	4		0		
No	9	13		4		
Smoking status			0.5911		0.46	0.75
Never smoker	5	9		3		
Previous smoker	2	4		1		
Current smoker	3	2		0		
Unknown	0	2		0		
Vaginal pH			0.0053		>0.99	0.07
Normal	6	1		2		
High	4	15		2		
Unknown	0	1		0		
Histotype (%)						
Endometrioid adenocarcinoma	-	11 (64.7)		-		
Serous adenocarcinoma	-	3 (17.6)		-		
Mucinous adenocarcinoma	-	1 (5.9)		-		
Squamous adenocarcinoma	-	1 (5.9)		-		
Carcinosarcoma	-	1 (5.9)		-		
Grade (%)						
Grade 1/Grade 2	-	13 (76.5)		-		
Grade 3	-	4 (23.5)		-		
Stage (%)						
I	-	13 (76.5)		-		
III/IV	-	4 (23.5)		-		

*BMI* body mass index, *IQR* interquartile range

### Microbiome characterization

**Fig. 1:**
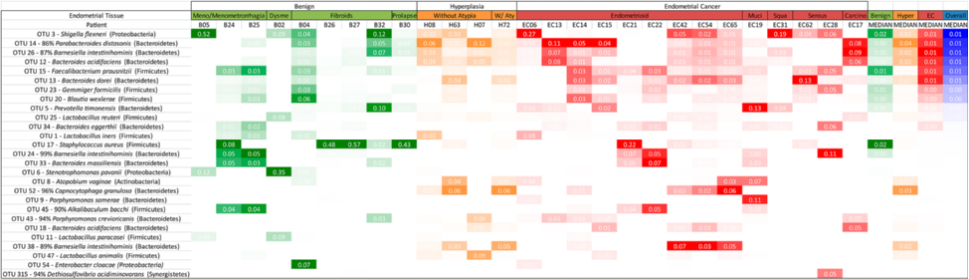
Endometrial microbiome across cohorts. Only taxa present at a minimum of 5 % relative frequency in at least one participant are shown for graphical clarity. Taxa *color scheme* reflects abundance relative to each patient (darker coloration represents higher abundance). *Meno/Menometrorrhagia* menorrhagia/menometrorrhagia, *Dysme* dysmenorrhagia/pelvic pain, *W/Aty* with atypia, *Muci* mucinous, *Squa* squamous, *Carcino* carcinosarcoma, *Hyper* hyperplasia

**Fig. 2:**
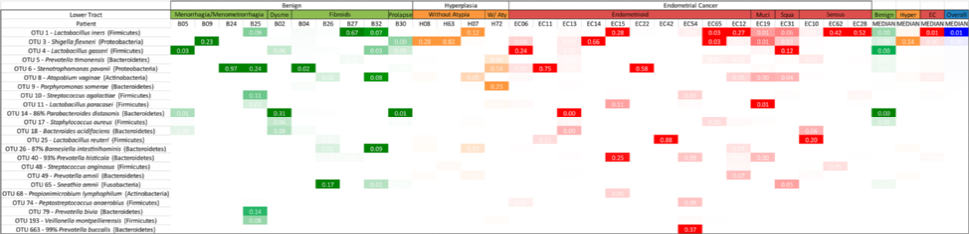
Vaginal/cervical (lower tract) microbiome across cohorts. Only taxa present at a minimum of 5 % relative frequency in at least one participant are shown for graphical clarity. Taxa *color scheme* reflects abundance relative to each patient (darker coloration represents higher abundance). *Dysme* dysmenorrhagia/pelvic pain, *W/Aty* with atypia, *Muci* mucinous, *Squa* squamous, *Hyper* hyperplasia

**Fig. 3:**
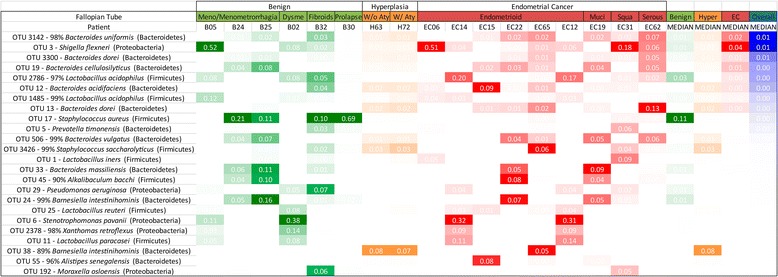
Fallopian tube microbiome across cohorts. Only taxa present at a minimum of 5 % relative frequency in at least one participant are shown for graphical clarity. Taxa *color scheme* reflects abundance relative to each patient (darker coloration represents higher abundance). *Meno/Menometrorrhagia* menorrhagia/menometrorrhagia, *Dysme* dysmenorrhagia/pelvic pain, *W/o Aty* without atypia, *W/Aty* with atypia, *Muci* mucinous, *Squa* squamous, *Hyper* hyperplasia

**Fig. 4:**
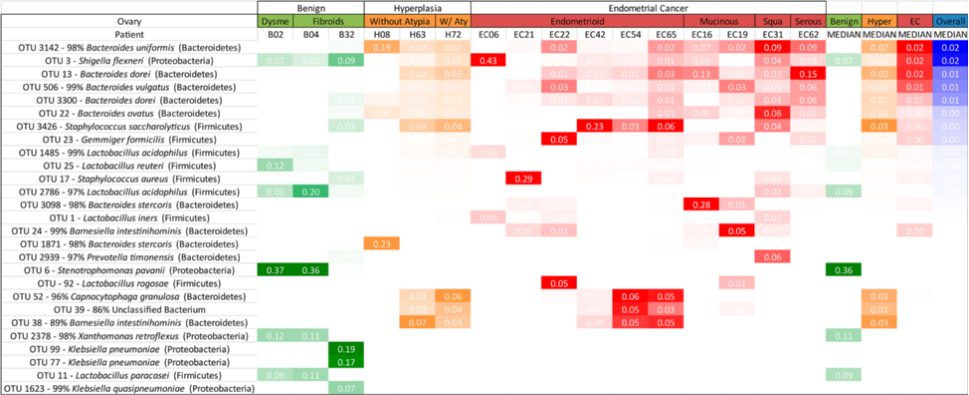
Ovarian microbiome across cohorts. Only taxa present at a minimum of 5 % relative frequency in at least one participant are shown for graphical clarity. Taxa *color scheme* reflects abundance relative to each patient (darker coloration represents higher abundance). *Dysme* dysmenorrhagia/pelvic pain, *W/Aty* with atypia, *Squa* squamous, *Hyper* hyperplasia

In order to characterize the microbiome of the patients we collected vaginal and cervical samples (lower genital tract) in the operating room and endometrial, Fallopian, and ovarian samples in the pathology laboratory (collection details are provided in the “Methods” section). The deep-sequencing of the V3-V5 16S rDNA region of all 238 collected samples resulted in the identification of 3545 OTUs. The endometrial microbiome was dominated by *Shigella* and *Barnesiella*, with *Staphylococcus*, *Blautia*, and *Parabacteroides* particularly relevant in the benign cohort and *Bacteroides* and *Faecalibacterium* more relevant in the endometrial cancer cohort (Fig. 1). The uterine microbiome results are consistent with the very limited number of studies that have assessed the human microbiome composition through culture-based methods, where *Escherichia*, *Streptococcus*, *Staphylococcus*, and *Enterococcus* were found to be the predominant taxa in women with chronic endometritis and dysfunctional bleeding [21]. The very recent 16S rDNA assessment of the uterine microbiome via transcervical collection is also consistent with *Bacteroides* being a dominant uterine taxa [22]. In the lower genital tract (vagina and cervix), *Prevotella* and *Lactobacillus* were the dominant taxa, with *Stenotrophomonas* and *Shigella* more characteristic in the benign cohort and *Porphyromonas* more common in the endometrial cancer cohort (Fig. 2). These results are also consistent with the pre-menopausal and post-menopausal profiles reported by others [23, 24], with the exception of *Stenotrophomonas.* Because our benign population is not gynecologically healthy, but instead presented with a variety of conditions (pelvic pain, abnormal bleeding, fibroids, and prolapse), it is possible that *Stenotrophomonas* may be more prominent in this patient population than in an asymptomatic group of participants. Although it is also possible that this could be the result of contamination, we did not find this taxon to be prominent in our controls (Additional file 1). It is therefore unlikely that this is the case. In the Fallopian tubes, *Shigella* and *Bacteroides* were the most dominant taxa, with *Staphylococcus*, *Lactobacillus*, *Barnesiella*, and *Pseudomonas* commonly appearing in the benign cohort (Fig. 3). In the ovaries, *Stenotrophomas*, *Xanthomonas*, and *Lactobacillus* dominated the benign cohort, while *Bacteroides* dominated the endometrial cancer cohort (Fig. 4). There is no current literature on the human microbiome composition of Fallopian tubes or ovaries.

### Organ microbiome correlation

We first started by assessing if the microbiomes between the different organs were correlated. For instance, whether the vaginal microbiome of a given patient resembled the uterine microbiome of that particular patient more than the uterine microbiome of any other patient. The results showed a very significant correlation between all organs based on a distance-based permutation test (See “Methods” and Table 2). The correlation was also significant, though to a lesser degree, for the stool samples when compared to all organs. The correlation structure held for both benign and cancer cohorts (Additional file 2). Genus level analysis revealed several genera that were significantly shared between the lower genital tract and uterus (Additional file 3). These results are indicative of an overall host specific microbiome effect (host selection effect) and/or transfer of microbiomes across the different organs (microbial movement across organs). The correlation between organs also suggests a potential gain in statistical power by a combined analysis. We thus performed both combined (uterus + lower genital tract) and separate analyses when assessing the microbiota between different disease states.

**Table 2:** Organ correlation *p* values based on Bray-Curtis distance-based permutation tests

	Fallopian	Lower	Ovary	Stool	Uterus
Fallopian	0	0.001	0.001	0.005	0.001
Lower	0.001	0	0.001	0.014	0.001
Ovary	0.001	0.001	0	0.022	0.001
Stool	0.005	0.014	0.022	0	0.013
Uterus	0.001	0.001	0.001	0.013	0

### Overall microbiome structure difference between benign, hyperplasia, and endometrial cancer

**Fig. 5:**
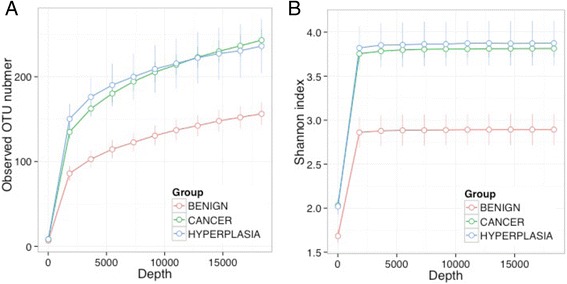
α-diversity comparison between different disease states in the endometrial microbiome. *Error bars* represent the standard errors. **a** Observed OTU number. **b** Shannon index

**Fig. 6:**
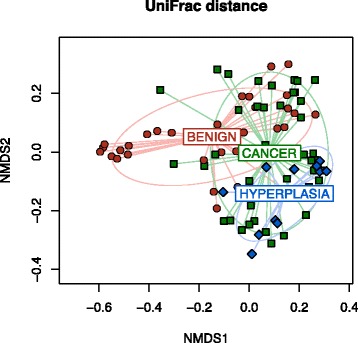
*Ordination plot* based on unweighted UniFrac distance depicting the relationship between different disease states. Each *point* represents a sample and is *colored* by sample group

We first compared the overall microbiota structure between disease states by investigating the α-diversity and β-diversity. The α-diversity (number of observed OTUs and Shannon index) in the cancer cohort was significantly higher than in the benign cohort (*p* = 0.003 and 0.01 for the two α-diversity metrics, LME) and the difference was much stronger in uterus (*p* = 0.03 and 0.01, Fig. 5) than in the lower genital tract (*p* = 0.17 and 0.31, Additional file 4). The endometrial α-diversity of the hyperplasia cohort was similar to the cancer cohort and was also significantly higher than the benign cohort (*p* = 0.07 and 0.04, Fig. 5). β-diversity analysis revealed a significant difference in the overall microbiota structure between the three cohorts (*p* = 0.01, unweighted UniFrac, PERMANOVA, Fig. 6). Consistent with the α-diversity analysis, the difference was mainly observed in the uterus (*p* = 0.05 and 0.11 for uterus and lower genital tract, unweighted UniFrac). We next conducted pairwise comparisons using the endometrial samples. The endometrial microbiome of both endometrial cancer and hyperplasia cohorts displayed some level of difference from the benign cohort (*p* = 0.09 and 0.07, unweighted UniFrac). In contrast, the hyperplasia cohort was not distinguishable from the endometrial cancer cohort (*p* = 0.23, unweighted UniFrac) (Fig. 6). Comparison of the distance between the benign and hyperplasia cohort to the distance between cancer and hyperplasia cohort reveals that hyperplasia is closer to the cancer cohort (*p* = 0.05, unweighted UniFrac, permutation test; Additional file 5). Interestingly, the distance between the benign and hyperplasia cohort is also significantly larger than that between the benign and cancer cohort (*p* = 0.05, unweighted UniFrac, Additional file 5). Because endometrial hyperplasia can be a clinical precursor to endometrial cancer, and the uterine microbiome of the four patients diagnosed with endometrial hyperplasia is distinct from the benign cohort and presents some but not complete clustering with an endometrial cancer subgroup, we removed these patients from the primary analysis. This allowed us to compare the benign and endometrial cancer cohorts without the impact of the hyperplasia cases. These were later introduced in a secondary analysis.

The dataset also contains Fallopian and ovarian samples. We therefore tested the microbiota difference between the benign and cancer cohorts for these two organs. Interestingly, we identified a significant difference for the ovaries (*p* = 0.003, unweighted UniFrac, Additional file 6) suggesting a microbiome connection between the ovarian microniche and endometrial cancer presence/absence.

### Endometrial cancer microbiome signature

**Fig. 7:**
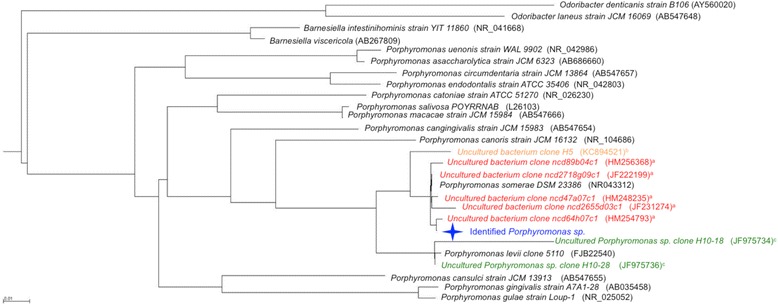
Maximum likelihood *phylogenetic tree* of the V3-V5 16S rDNA region of the recovered *Porphyromonas* sp. ^a^ Recovered from children with atopic dermatitis. ^b^ Recovered from buffaloes with postpartum endometritis. ^c^ Recovered from Holstein dairy cows with postpartum metritis. Produced with FASTTREE

After the overall microbiome assessment, we performed taxa analysis to determine whether the benign and endometrial cancer cohort displayed differential microbiota. We first performed a combined analysis pooling the samples from both the uterus and lower genital tract. At the genus level there were 12 taxa significantly enriched in the endometrial cancer cohort (Table 3 and Additional file 7, q < 0.10). When we further inquired at a finer level (OTU), we found eight OTUs significantly associated with endometrial cancer (Table 4, q < 0.05). OTU 8 (*Atopobium* sp.) and OTU 9 (*Porphyromonas* sp.) became of particular relevance since they were pervasive across samples recovered from endometrial cancer patients and largely absent from the samples recovered from patients in the benign cohort. The *Atopobium* V3-V5 16S rDNA signature matches (100 %) that of *Atopobium vaginae*, a well-known vaginal pathogen [25]. The *Porphyromonas* signature is a close match (99 % sequence identity) to *Porphyromonas somerae* (Fig. 7), a described pathogen recovered from soft tissue and bone infections [26]. Separate analyses of endometrial and lower genital tract samples revealed a high concordance of the identified genera from the pooled analysis, indicating that both uterine and lower genital tract microbiota may be associated with cancer diagnosis (Table 3).

**Table 3:** Significant bacterial genera between benign and endometrial cancer cohorts

Combined q < 0.10	Value	Standard error	Degrees of freedom	t value	*p* value	q value	Test
Firmicutes; Anaerostipes	3.4	0.795	25	4.3	0.0002	0.017	Presence/absence
Firmicutes; ph2	3.1	0.829	25	3.7	0.0010	0.031	Presence/absence
Spirochaetes; Treponema	3.9	1.066	25	3.7	0.0011	0.031	Presence/absence
Actinobacteria; Atopobium	2.5	0.707	25	3.5	0.0017	0.036	Presence/absence
Bacteroidetes; Bacteroides	1.1	0.332	25	3.3	0.0026	0.044	Counts
Proteobacteria; Arthrospira	3.6	1.150	25	3.1	0.0044	0.062	Presence/absence
Firmicutes; Dialister	1.2	0.405	25	3.0	0.0061	0.073	Presence/absence
Firmicutes; Peptoniphilus	1.4	0.494	25	2.9	0.0074	0.075	Presence/absence
Firmicutes; 1-68	1.3	0.465	25	2.9	0.0080	0.075	Presence/absence
Firmicutes; Ruminococcus	0.9	0.319	25	2.8	0.0109	0.082	Counts
Bacteroidetes; Porphyromonas	1.8	0.664	25	2.7	0.0111	0.082	Presence/absence
Firmicutes; Anaerotruncus	1.3	0.477	25	2.7	0.0117	0.082	Presence/absence

Significant bacterial genera between benign and endometrial cancer cohorts in the vaginal, cervical, and endometrial microbiome as determined by generalized mixed effect model (FDR q < 0.10). All genera are enriched in the endometrial cancer cohort

**Table 4:** Significant bacterial operational taxonomic units (OTUs) between benign and endometrial cohorts

Combined q < 0.05	Value	Standard error	Degrees of freedom	t value	*p* value	q value	Test
OTU 107: Firmicutes; Anaerostipes	3.3	0.725	25	4.6	0.0001	0.014	Presence/absence
OTU 143: Firmicutes; Ruminococcus	3.1	0.738	25	4.2	0.0003	0.019	Presence/absence
OTU 8: Actinobacteria; Atopobium	2.5	0.603	25	4.1	0.0004	0.019	Presence/absence
OTU 3197: Bacteroidetes; Bacteroides	2.5	0.655	25	3.9	0.0007	0.024	Presence/absence
OTU 3213: Bacteroidetes; Bacteroides	1.7	0.499	25	3.5	0.0018	0.043	Presence/absence
OTU 9: Bacteroidetes; Porphyromonas	1.9	0.554	25	3.4	0.0021	0.043	Presence/absence
OTU 138: Bacteroidetes; Bacteroides	1.7	0.517	25	3.4	0.0024	0.043	Presence/absence
OTU 181: Firmicutes; Dialister	2.0	0.585	25	3.4	0.0025	0.043	Presence/absence

Significant bacterial OTUs between benign and endometrial cohorts in the vaginal, cervical, and endometrial microbiome as determined by mixed effect model (FDR q < 0.05). All OTUs shown in the table are enriched (more prevalent) in the endometrial cancer cohort

### Vaginal pH and endometrial cancer

Vaginal pH was significantly correlated with an endometrial cancer diagnosis (*p* = 0.0053), with endometrial cancer patients typically displaying a high vaginal pH (>4.5). However, the vaginal pH is known to raise in approximately 95 % of postmenopausal women [27] due to physiological and microbiological changes [28]. Therefore, the correlation between endometrial cancer and high vaginal pH could not be detangled from age effects alone. Nevertheless, we were able to determine that the microbiome pH effects were independent of the microbiome disease effects in the uterus since the vaginal pH level was not significantly correlated with the uterine microbiome (*p* = 0.22 and 0.29, unweighted and weighted UniFrac, PERMANOVA), indicating that they can be used as distinct factors.

### Lower tract microbiome association with endometrial cancer

**Fig. 8:**
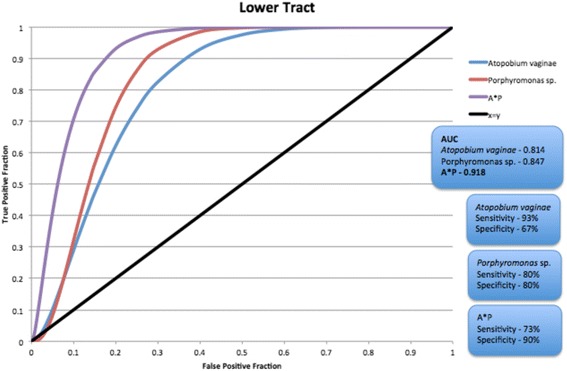
*ROC curve* for *Atopobium vaginae* and *Porphyromonas* sp. presence in the lower reproductive tract (vagina/cervix) and disease status (benign vs. endometrial cancer)

In the lower genital tract, the association of *Atopobium vaginae* and the identified *Porphyromonas* sp. with a diagnosis of endometrial cancer has a sensitivity of 73–93 %, and specificity of 67–90 % (Fig. 8). The sensitivity is improved if the vaginal pH is factored in, although specificity is decreased (Table 5; sensitivity – 100 %, specificity – 60 %).

**Table 5:** Correlation between the detection of *Atopobium vaginae* and *Porphyromonas sp.* and vaginal pH with disease status

Benign	pH	*A. vaginae*	*Porphyromonas* sp.	A*P	A or P + pH
B02	Normal	Positive	Negative	Negative	Negative
B04	Normal	Negative	Negative	Negative	Negative
B05	Normal	Negative	Negative	Negative	Negative
B09	Normal	Negative	Negative	Negative	Negative
B24	Normal	Negative	Negative	Negative	Negative
B25	High	Positive	Negative	Negative	Positive
B26	High	Negative	Positive	Negative	Positive
B27	High	Positive	Positive	Positive	Positive
B30	Normal	Negative	Negative	Negative	Negative
B32	High	Positive	Negative	Negative	Positive
Cancer	pH	*A. vaginae*	*Porphyromonas* sp.	A*P	A or P + pH
EC06	High	Positive	Positive	Positive	Positive
EC10	High	Positive	Positive	Positive	Positive
EC11	High	Positive	Positive	Positive	Positive
EC12	High	Positive	Positive	Positive	Positive
EC13	Normal	Positive	Positive	Positive	Positive
EC14	Normal	Positive	Positive	Positive	Positive
EC15	Normal	Positive	Positive	Positive	Positive
EC19	High	Positive	Positive	Positive	Positive
EC22	Normal	Positive	Positive	Positive	Positive
EC28	High	Positive	Positive	Positive	Positive
EC31	High	Positive	Negative	Negative	Positive
EC42	High	Positive	Negative	Negative	Positive
EC54	High	Positive	Positive	Positive	Positive
EC62	NA	Negative	Positive	Negative	NA
EC65	High	Positive	Negative	Negative	Positive
Hyperplasia	pH	*A. vaginae*	*Porphyromonas* sp.	A*P	A or P + pH
H07	High	Negative	Negative	Negative	Negative
H08	High	Negative	Negative	Negative	Negative
H63	Normal	Negative	Positive	Negative	Negative
H72	Normal	Positive	Negative	Negative	Negative

Correlation between the vaginal/cervical detection of *Atopobium vaginae* and *Porphyromonas sp.* (positive = detected/negative = undetected) and vaginal pH measurement (normal ≤ 4.5; high > 5) with disease status

### Endometrial hyperplasia microbiome

We had four patients with a final diagnosis of endometrial hyperplasia, which is a known endometrial cancer precursor, in particular in the case of complex hyperplasia with atypia. Three of our patients had simple hyperplasia without atypia (H07, H08, and H63) and one had complex hyperplasia with atypia (H72). Interestingly, the *Atopobium vaginae* and the *Porphyromonas* sp. presence/absence profile of the vaginal microbiome of these four patients more closely resembled a benign microbiome signature (Table 5), while the uterine microbiome signature of two of them (H63 and H72) were closer to an endometrial cancer signature.

### Snapshots of progression

**Fig. 9:**
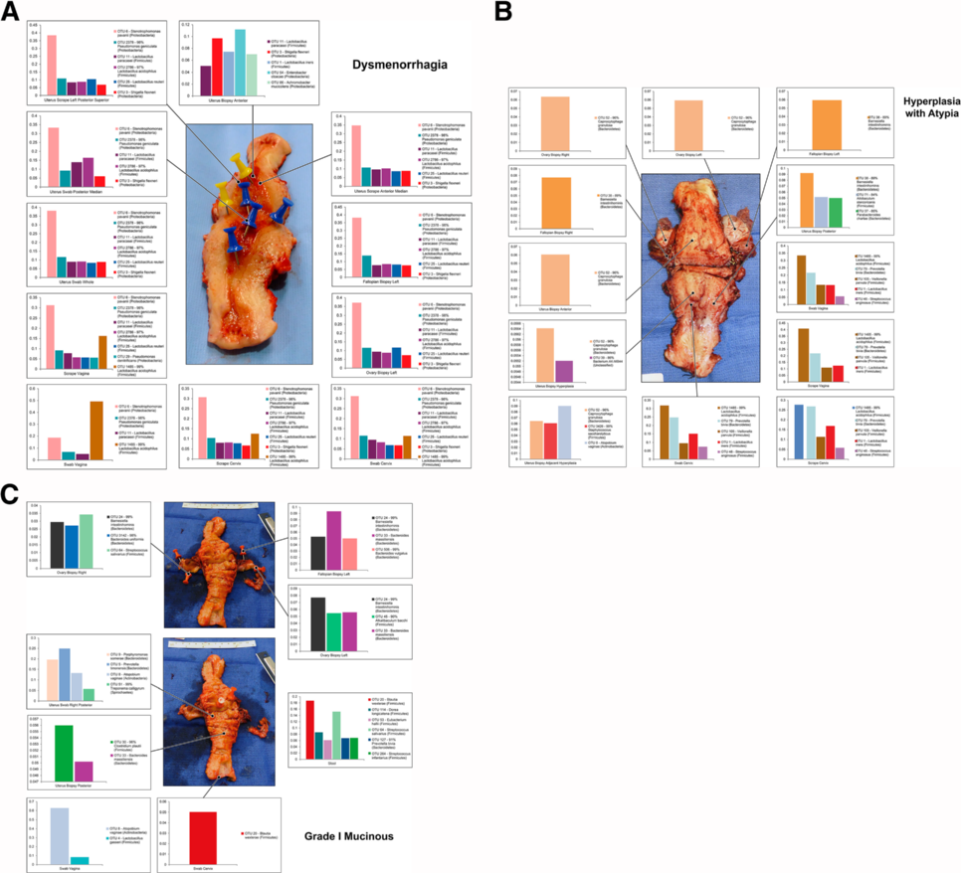
Example collections. Only taxa present at more than 5 % relative frequency per sample are shown for graphical clarity. **a** Patient B02. **b** Patient H72. **c** Patient EC19

The correlation and variation between the microbiomes recovered is illustrated in the snapshots, which demonstrate the variable microbiome landscape within and between patients (Fig. 9). We were able to successfully amplify bacterial DNA from 94 % of the lower genital tract samples (vaginal/cervical), 87 % of uterine samples, 50 % of the Fallopian, 61 % of ovarian, 29 % of urine, and 17 % of peritoneal or ascites samples. This progression is likely representative of the bacterial burden in the different body sites.

## Discussion

Here we present a pilot high-throughput microbiome assessment of the female reproductive tract of patients diagnosed with a variety of benign uterine conditions warranting a hysterectomy (abnormal bleeding, fibroids, uterine prolapse, and pelvic pain), endometrial hyperplasia (with and without atypia), and an endometrial cancer diagnosis (endometrioid, mucinous, serous, squamous, and carcinosarcoma). The dominant taxa in the vaginal and cervical (lower tract) microbiome were *Prevotella* and *Lactobacillus*, which is consistent with current vaginal microbiome literature [23]. The dominant bacteria in the uterine microbiome were *Shigella* and *Barnesiella*, which is also consistent with the existent culture-based literature of the uterine microbial composition in women with endometritis and abnormal bleeding [21]. However, in contrast with our data, these authors reported low concordance between the vaginal and uterine bacteria. Despite the low number of patients in our study (n = 31), the microbiome correlation between all organs (vagina/cervix, uterus, Fallopian tubes, ovaries) was very significant, including the stool recovered from each patient. We interpret these results to indicate that there is a strong selective host effect on the microbiome and/or that there is movement or transfer of microorganisms across the different body sites. Since the samples were not collected simultaneously or in the same location or by the same personnel, we do not believe this correlation to be the result of an artifact. In addition, the stool samples were collected by the patient and processed separately, further supporting the veracity of the correlation. The microbiome organ correlations were equally significant for benign and endometrial cancer patients. Because we do not have healthy asymptomatic patients in this study we cannot assess whether this correlation is generally present or if it may be indicative of a diseased status in itself. We were unable to amplify a positive bacterial signal in about 40–50 % of all Fallopian and ovarian tissue biopsies collected. We conjecture that these results indicate a very low bacterial load (below detection) rather than a truly sterile environment. Further advancements in our ability to detect and amplify bacterial DNA from tissue samples should improve this success rate.

Our results indicate that endometrial hyperplasia can be distinguished from a benign uterine condition based on its microbiome structure, suggesting either a microbiome role in the early phases of cellular transformation or a notorious response to physiologic or chemical gradient shifts within the host’s cellular microenvironment. This structural differentiation is not apparent between hyperplasia and endometrial cancer patients or between benign and endometrial cancer patients. This could be indicative of a transient microbial ecological disturbance that is later normalized to a new equilibrium state, overall closer to the starting point.

Our results suggest that the detection of *A. vaginae* and the identified *Porphyromonas* sp. in the gynecologic tract is associated with the presence of endometrial cancer, especially if combined with a high vaginal pH (>4.5). Though cause and effect cannot be discerned from association alone, examination of the hyperplasia cases shows that although these microbes are absent from the lower tract, they are present in half the cases in the uterus, supporting an early disease role for these microbes. *A. vaginae* has been increasingly recognized as a prominent gynecologic and obstetric pathogen, being positively associated with Nugent scores and bacterial vaginosis [25], intrauterine infections [29], and other invasive infections of the female genital tract [30]. While we did not anticipate finding *Atopobium* to be associated with endometrial cancer, we provided all patients with a comprehensive questionnaire about present and past gynecologic and obstetric diseases or conditions. One question directly asked if the patient had a current or past diagnosis of bacterial vaginosis. Only one patient (H72), a patient with hyperplasia, declared having had bacterial vaginosis in the past (more than six months away from present time). Through verification of the medical records, which is allowed by our institutional IRB, no additional patients had a previous diagnosis of bacterial vaginosis in their medical record. While bacterial vaginosis is a condition difficult to diagnose and not always medically reported, we believe we did all we could reasonably do to assess the prevalence of this disease in our study population. Given that only one patient indicated a past occurrence of bacterial vaginosis, we do not have evidence that this disease impacted our results significantly or differently among our benign and study cohort. While the specific *Porphyromonas* sp. has yet to be characterized in the literature, the association of members of the *Porphyromonas* genus with cancers has been recently verified. *Porphyromonas gingivalis* has been found to be an accurate biomarker for risk of death due to orodigestive cancer, independently of periodontal disease [31], and the carriage of *Porphyromonas* species has also been found to be associated with colorectal cancer participants [10]. Given the correlation of these two microorganisms with the disease along with their association with other pathologies, it is possible that they are involved in the etiology or aggravation of conditions leading up to the development of endometrial cancer. Based on the documented association of *A. vaginae* [32] with bacterial vaginosis, it is possible that this microbe causes a chronic inflammatory profile that eventually leads to local immune dysregulation and facilitates intracellular infection by *Porphyromonas* species. *Porphyromonas* species have been found intracellularly [33] and it is conceivable that the microorganism we found is capable of disrupting normal cell regulatory functions that may eventually lead to a carcinogenic trigger. The trigger could then be reinforced by the anoxic microenvironment fomented by these microorganisms. We believe this to be a hypothesis worthy of mechanistic investigation.

Our study is limited by a small sample size, due to the technical difficulty of collecting these specimens in real time in the operating room and in the pathology laboratory within a sterile field while guaranteeing that the diagnosis and diagnostic time is not impact by our research procedures. While sample size is always a valid concern, based on the microbiome-based power calculation, the current sample size is powered to detect a relatively large overall effect: 90 % power for an ω^2^ = 0.04, unweighted UniFrac, an effect size similar to that of antibiotics [34]. Even at this small sample size, we were still able to identify significant microbiome differences between disease states and identify differential abundant taxa after multiple testing correction. Though the increased power may be partially due to the inclusion of multiple replicates for each participant and the use of sophisticated statistical models to address the correlation between replicates and thus reduce the sampling error, these significant results nevertheless indicate that there is a large difference between benign and cancer states.

We investigated potential sources of confounding in this comparative study. The study involved slightly different methods of sampling the microbiota including variation in bacterial DNA enrichment (used to separate bacterial DNA from human DNA in tissue samples), collection type (swab versus scrape versus biopsy), and sampling position (posterior versus superior). Based on marginal PERMANOVA tests, we were able to detect significant effects of bacterial DNA enrichment method and collection type in profiling the microbiota (*p* < 0.001, unweighted UniFrac) while the sampling position was not significant (*p* = 0.28 and 0.67, unweighted and weighted UniFrac). However, these technical variables were not true confounders in this comparative study due to roughly equal proportions of different sampling methods in both benign and cancer cohorts (*p* > 0.3, Fisher’s exact test). In fact, if these technical variables were adjusted in the model, we achieved a similar level of statistical significance in testing the microbiota difference between the two cohorts (data not shown). We thus ruled out the potential confounding effects of these technical variables. Among the demographical and clinical variables, age, BMI, vaginal pH level, menopausal status, and history of hypertension were potential confounders, which had different distributions in the benign and cancer cohorts (Table 1). Marginal PERMANOVA tests on the uterus samples revealed that these variables had less significant effects on the endometrial microbiota than the cohort effect (Additional file 8), indicating that the observed difference could not be completely explained by these potential confounders. However, a larger sample study may be needed to disentangle these confounding effects with confidence. In order to specifically address concerns that the observed findings could be impacted by the age differential between our benign and cancer cohorts, we performed a subset analysis where we extracted patients in the age range of 48–60 years with both a cancer and benign diagnosis. We repeated the analysis with this age-matched subset and we still observed the same trend for both *A. vaginae* and *Porphyromonas*, though less significant due to the reduction in the sample size (6 cancer versus 5 benign, Additional file 9). Although age differential is a direct reflection of the patient populations, enrollment targeted efforts will be made in future studies to decrease this gap.

Lastly, while our enrollment exclusion and inclusion criteria did not specify nor exclude any ethnic or racial description, our study population is entirely Caucasian. Although this is a representative reflection of the composition of our patient population at Mayo Clinic, Rochester, MN, it is not representative of the country’s demographics. In future studies we will seek an ethnically diverse patient population to investigate whether our results extend to other populations.

### Future directions

Extending this study to a larger number of patients will allow for the verification of the findings and increase the statistical power. Culturing the identified *Porphyromonas* sp. and investigating its effects on endometrial cells and their immunological pro-inflammatory profile response, especially in the co-presence of *A. vaginae*, is warranted. Because of the modifiable nature of the microbiome, these findings also hold promise to endometrial cancer prevention.

## Conclusions

We found a distinct microbiome signature in patients with endometrial cancer and hyperplasia. We have shown that in our study population the detection of *A. vaginae* and the identified *Porphyromonas* sp. in the gynecologic tract is associated with the presence of endometrial cancer, especially if combined with a high vaginal pH (>4.5). These findings provide important insights into the etiology or manifestation of the disease with broad implications for biomarker development in the early detection of and screening for endometrial cancer.

## Additional files

Additional file 1: Sequencing results from controls. A total of 14 controls were performed, with five of them not retrieving any sequence reads. sV1, sV2 – V-shaped scrape with Microbiome Enrichment Nebnext kit; sP1, sP2 – Pap scrape with Microbiome Enrichment Nebnext kit; sT1, sT2 – TE buffer with Microbiome Enrichment Nebnext kit; sS1 – Swab with Microbiome Enrichment Nebnext kit; sV1B, sV2B – V-shaped scrape. (XLSX 26 kb)
Additional file 2: Organ correlation with benign and endometrial cancer patients. (XLSX 43 kb)
Additional file 3: Genera correlation between lower tract and uterus. (XLSX 51 kb)
Additional file 4: α-diversity for the lower tract. **A** Observed OTU number. **B** Shannon index. (PDF 384 kb)
Additional file 5: Unweighted UniFrac distance between benign, hyperplasia, and endometrial cancer cohorts. (PDF 8 kb)
Additional file 6: Unweighted UniFrac distance for ovary between benign and endometrial cancer cohort. (PDF 9 kb)
Additional file 7: Genera significantly enriched in the endometrial cancer cohort. (PDF 11 kb)
Additional file 8: Confounder analysis. (XLSX 37 kb)
Additional file 9: Cohort age differential subset analysis. (XLS 33 kb)

## Abbreviations

AUC: Area under the curve
BMI: Body mass index
EDTA: Ethylenediaminetetraacetic acid
FDR: False discovery rate
HIF: Hypoxia-inducible factor
IM-TORNADO: Illinois-Mayo Taxon Operations for RNA Dataset Organization
IQR: Interquartile range
LB: Lysogeny broth
LME: Linear mixed effects model
MSI: Microsatellite instability
mTOR: Mammalian target of rapamycin
OTU: Operational taxonomic unit
PA: Pathologist’s assistant
PCR: Polymerase chain reaction
PI3K: Phosphoinositide 3-kinase
PTEN: Phosphatase and tensin homolog
ROC: Receiver operating characteristic
TE: Tris-EDTA buffer
