# Supplementary figures and images for: Potential contribution of the uterine microbiome in the development of endometrial cancer
Source: Genome Med. 2016 Nov 25;8:122. doi: 10.1186/s13073-016-0368-y (PMC5123330; doi:10.1186/s13073-016-0368-y)

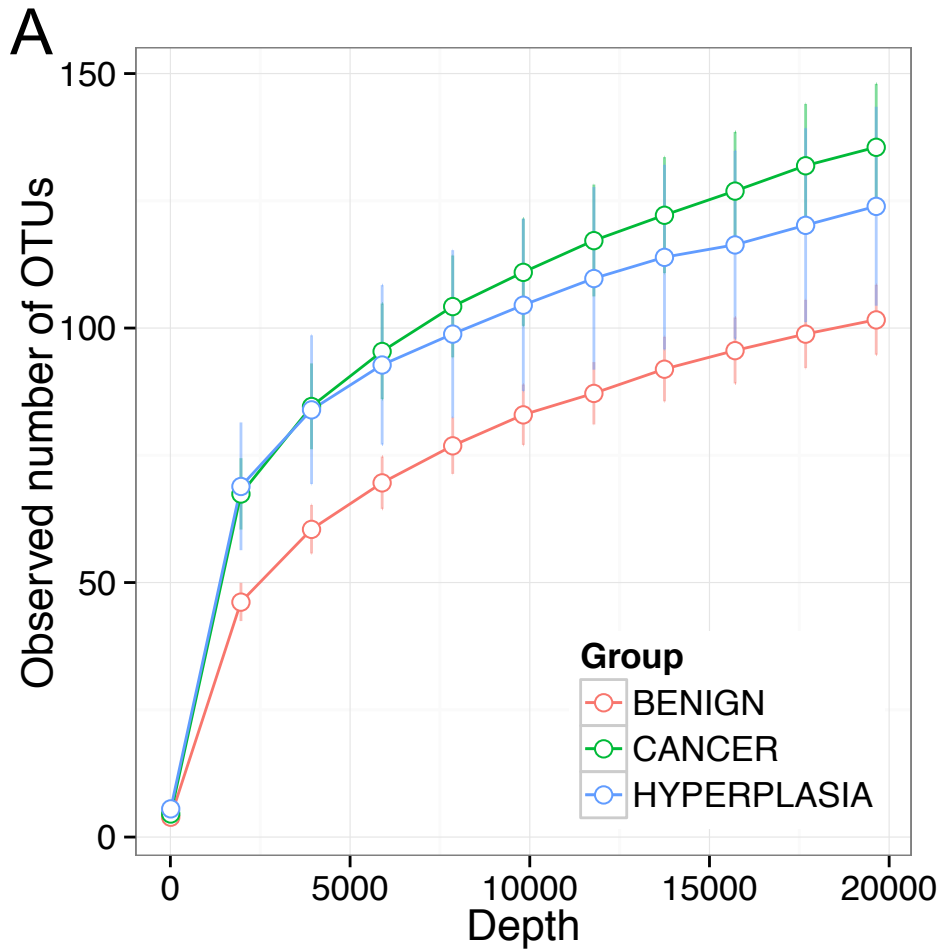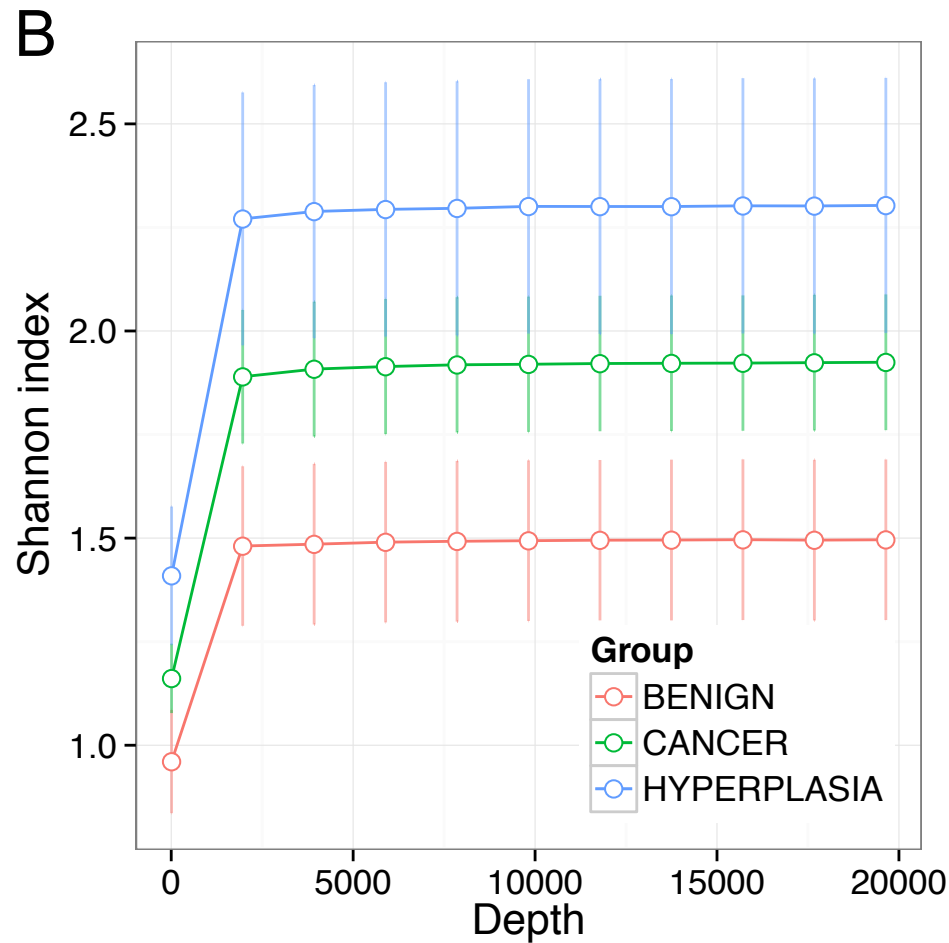

Supplement: Additional file 4: — α-diversity for the lower tract. A Observed OTU number. B Shannon index. (PDF 384 kb) [file 13073_2016_368_MOESM4_ESM.pdf]

UniFrac Distance

0.8

0.6

0.4

CANCER vs  
BENIGN

HYPERPLASIA vs  
BENIGN

HYPERPLASIA vs  
CANCER

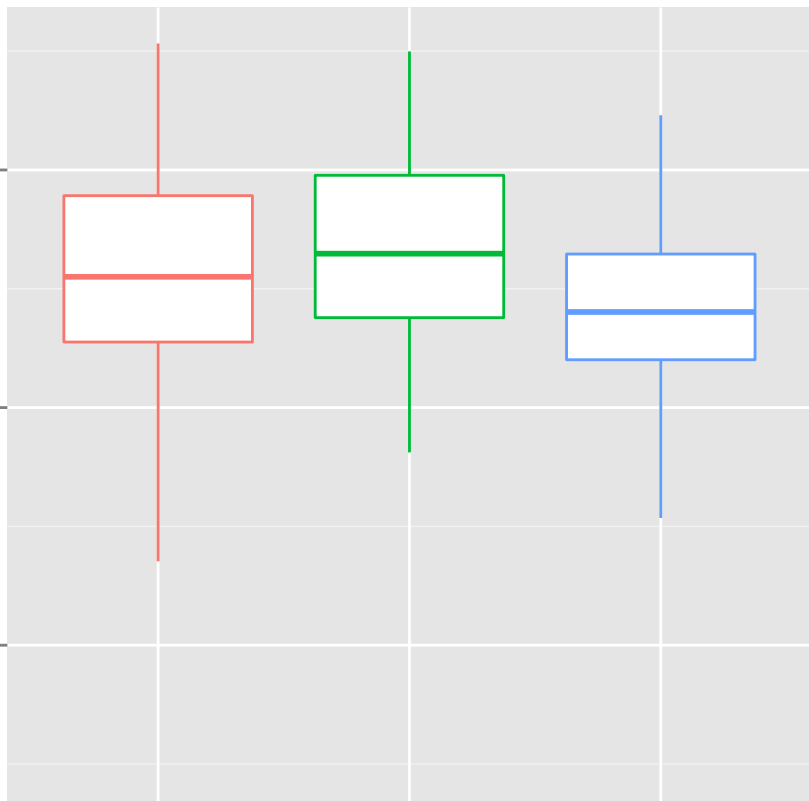

Supplement: Additional file 5: — Unweighted UniFrac distance between benign, hyperplasia, and endometrial cancer cohorts. (PDF 8 kb) [file 13073_2016_368_MOESM5_ESM.pdf]

# UniFrac distance

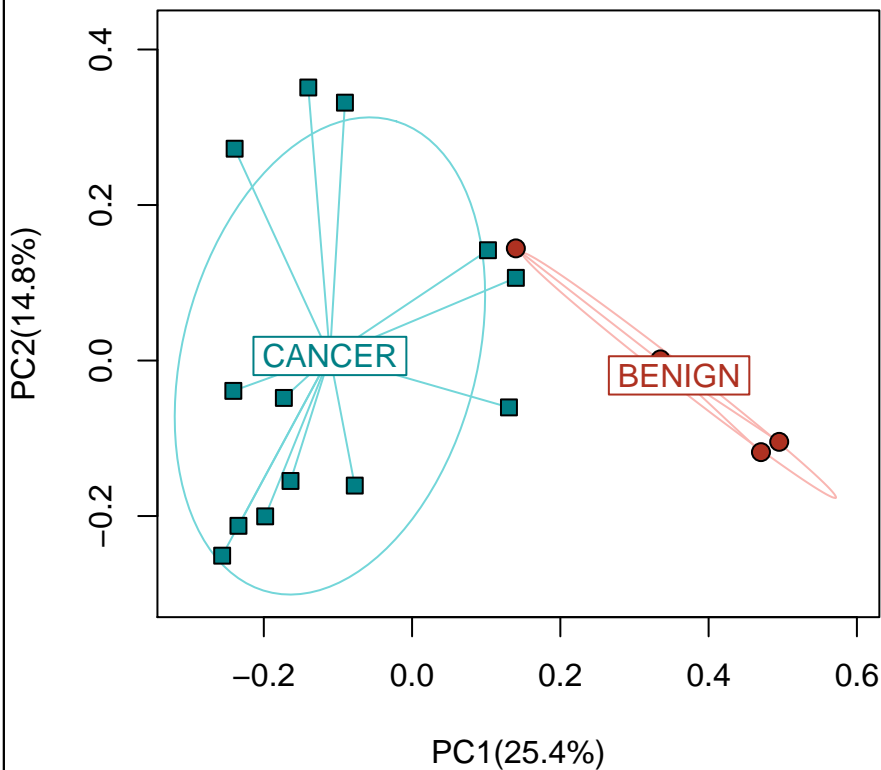

Supplement: Additional file 6: — Unweighted UniFrac distance for ovary between benign and endometrial cancer cohort. (PDF 9 kb) [file 13073_2016_368_MOESM6_ESM.pdf]

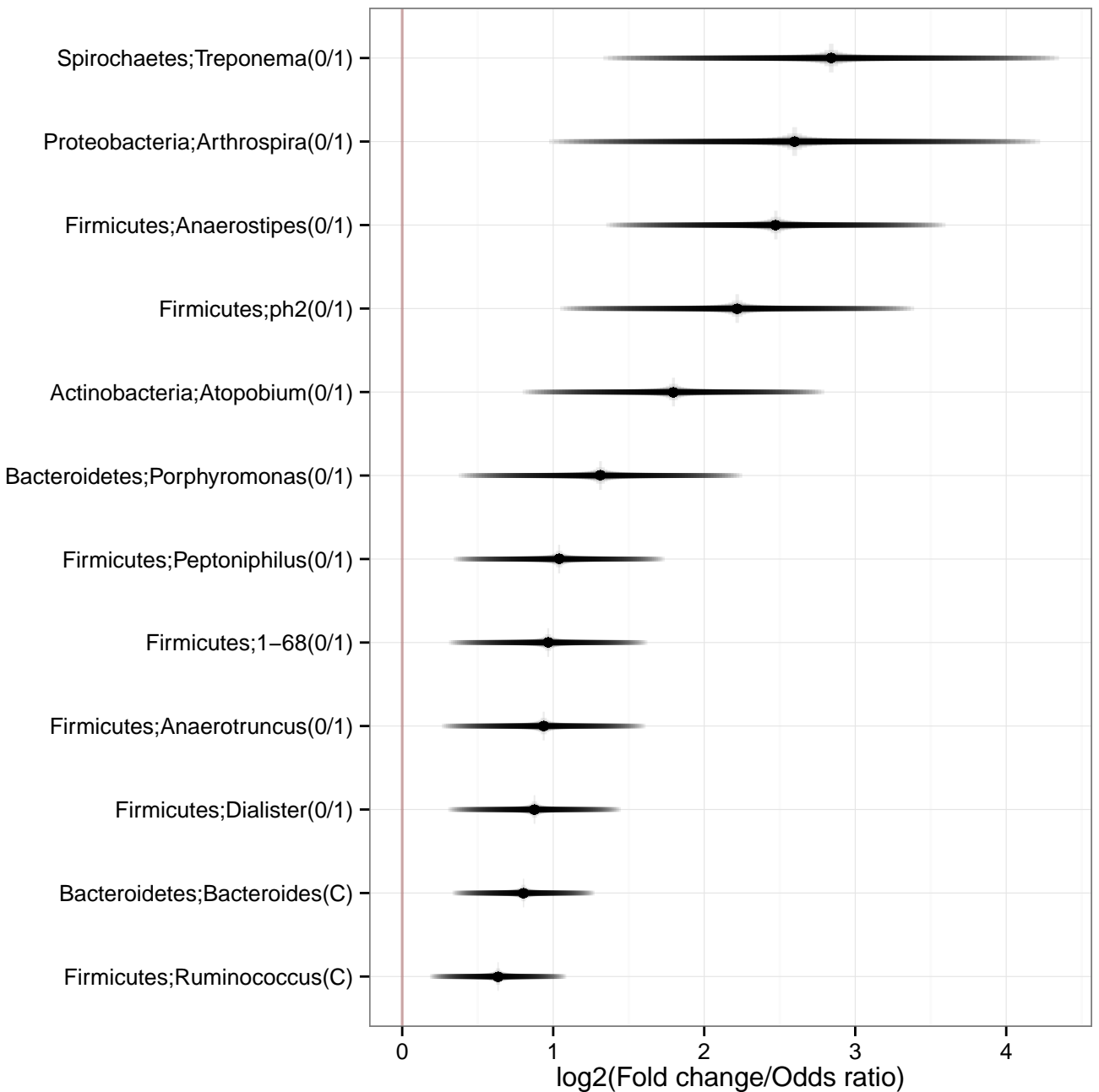

Supplement: Additional file 7: — Genera significantly enriched in the endometrial cancer cohort. (PDF 11 kb) [file 13073_2016_368_MOESM7_ESM.pdf]
